# Isolation of Consumptives

**Published:** 1899-05

**Authors:** 


THE consumptive will soon be as much dreaded as the leper, judging by the manner in which precautions are being taken to almost isolate him by both municipal and Federal government. We are heartily in favor of educating the public to the realization of the fact that it is dangerous for a healthy person to live constantly with a consumptive and, also, that the disease may be made contagious by the inhalation of dust from the dried sputum, but we do think that this poor, afflicted individual should be allowed some pleasures and comforts in life which are open to the public in general. It is bad enough to wander through life knowing that your days are numbered, but to be shunned by the public in every conceivable manner, should certainly be a thorn in the flesh. If things continue as they have been for the last two years, the consumptive will have to seek an isolated retreat in some uninhabited country and there pine his hours away in destitute silence. We see from the *British Medical Journal* that it has displayed great judgment when it says that "unless zeal in the cause be accompanied by wisdom and discretion, the consumptive will come to be regarded as something rather worse than a leper, and but little less dangerous to those with whom he may come in contact than a patient suffering from smallpox or the plague." It seems as if there is great travel from England to South Africa by poor, unfortunate consumptives in hopes thereby of recuperating the physical strength. Lately a great agitation has been started to prohibit consumptives from traveling on the steamers occupied by others not so afflicted, and such would have the traveling done on steamers run just for consumptives and no one else. Such action would certainly seem to be far-fatched, and if the idea was carried out we would soon have to have separate railroad trains, hotels, etc. We do not belive that phthisis is virulent enough to demand such drastic measures.

